# Clinical and analytical validation of FoundationOne Liquid CDx, a novel 324-Gene cfDNA-based comprehensive genomic profiling assay for cancers of solid tumor origin

**DOI:** 10.1371/journal.pone.0237802

**Published:** 2020-09-25

**Authors:** Ryan Woodhouse, Meijuan Li, Jason Hughes, David Delfosse, Joel Skoletsky, Pei Ma, Wei Meng, Ninad Dewal, Coren Milbury, Travis Clark, Amy Donahue, Dan Stover, Mark Kennedy, Jennifer Dacpano-Komansky, Christine Burns, Christine Vietz, Brian Alexander, Priti Hegde, Lucas Dennis

**Affiliations:** 1 Regulatory Affairs, Foundation Medicine, Inc, Cambridge, Massachusetts, United States of America; 2 Biometrics and Biomarkers, Foundation Medicine, Inc, Cambridge, Massachusetts, United States of America; 3 Computational Biology, Foundation Medicine, Inc, Cambridge, Massachusetts, United States of America; 4 Development Operations, Foundation Medicine, Inc, Cambridge, Massachusetts, United States of America; 5 Formerly Assay Development, Foundation Medicine, Inc, Cambridge, Massachusetts, United States of America; 6 Assay Development, Foundation Medicine, Inc, Cambridge, Massachusetts, United States of America; 7 Laboratory Operations, Foundation Medicine, Inc, Cambridge, Massachusetts, United States of America; 8 Medical Device Global Regulatory Affairs, Novartis Corporation, Cambridge, Massachusetts, United States of America; 9 Product Development, Foundation Medicine, Inc, Cambridge, Massachusetts, United States of America; 10 Clinical Development, Foundation Medicine, Inc, Cambridge, Massachusetts, United States of America; 11 Franchise Development, Foundation Medicine, Inc, Cambridge, Massachusetts, United States of America; University of California, San Francisco, UNITED STATES

## Abstract

As availability of precision therapies expands, a well-validated circulating cell-free DNA (cfDNA)-based comprehensive genomic profiling assay has the potential to provide considerable value as a complement to tissue-based testing to ensure potentially life-extending therapies are administered to patients most likely to benefit. Additional data supporting the clinical validity of cfDNA-based testing is necessary to inform optimal use of these assays in the clinic. The FoundationOne^®^Liquid CDx assay is a pan-cancer cfDNA-based comprehensive genomic profiling assay that was recently approved by FDA. Validation studies included >7,500 tests and >30,000 unique variants across >300 genes and >30 cancer types. Clinical validity results across multiple tumor types are presented. Additionally, results demonstrated a 95% limit of detection of 0.40% variant allele fraction for select substitutions and insertions/deletions, 0.37% variant allele fraction for select rearrangements, 21.7% tumor fraction for copy number amplifications, and 30.4% TF for copy number losses. The limit of detection for microsatellite instability and blood tumor mutational burden were also determined. The false positive variant rate was 0.013% (approximately 1 in 8,000). Reproducibility of variant calling was 99.59%. In comparison with an orthogonal method, an overall positive percent agreement of 96.3% and negative percent agreement of >99.9% was observed. These study results demonstrate that FoundationOne Liquid CDx accurately and reproducibly detects the major types of genomic alterations in addition to complex biomarkers such as microsatellite instability, blood tumor mutational burden, and tumor fraction. Critically, clinical validity data is presented across multiple cancer types.

## Introduction

As availability of precision therapies expands [1], there is an increasing reliance on genomic profiling assays to help identify the most relevant treatment options for advanced cancer patients [2–4]. Comprehensive genomic profiling (CGP) utilizes next generation sequencing (NGS) technology to examine entire exonic regions of cancer-relevant genes (in contrast to limited “hot spot” tests) for all tumor types, identifying the 4 main classes of genomic alterations: base substitutions (subs), insertions or deletions (indels), copy number alterations (CNAs), and gene rearrangements. Further, CGP assays can assess genomic alteration patterns across related genes in established cancer pathways to report complex biomarkers such as blood tumor mutational burden (bTMB) and microsatellite instability (MSI) to inform cancer treatment decisions using a single assay. Historically, CGP has utilized tumor tissue, although evaluable tumor tissue is not available for many patients [5–9]. with 38% of stage IV non-small cell lung cancer (NSCLC) patients in one single-center cohort study having insufficient quantity or quality of DNA for NGS. [7].

Often referred to as liquid biopsy assays, circulating cell-free DNA (cfDNA)-based assays, are a growing method for providing genomic profiling results to patients. There are a number of reasons that a liquid biopsy may be chosen in the clinical setting. For example, cfDNA-based testing is established for patients who are unable to provide evaluable tissue or when tissue quality or quantity is insufficient in a number of cancers [10–13]. Liquid biopsy assays may also offer a reduced time from sample to result as compared to tumor tissue assays due to the time required to provide a tumor sample for testing [14,15]. Additionally, due to intratumor heterogeneity, a tumor biopsy may represent a small sample of the overall tumor cell population, a limitation that can potentially be overcome with liquid biopsy [10, 16–18]. Tissue-based CGP has been shown to have improved clinical value compared to non-CGP testing, and liquid testing will likely add value based on its ability to find complementary information and provide biomarker results for patients unable to receive tissue testing [19].

Analytical validity of an NGS assay refers to how well the test identifies a particular genetic characteristic, such as a genomic alteration or genomic signature [20]. Analytical validation is important to demonstrate that a test accurately and reliably detects genomic alterations present in a sample. Clinical validity refers to the relationship between a genomic variant and the presence or absence of a specific disease, while clinical utility refers to the correlation of test results with improved health outcomes [20]. Clinical validation is critical to evaluate the correlation of test results with health outcomes. The US FDA requires robust analytical and clinical validation, above and beyond the validation standards set by Clinical Laboratory Improvement Amendments (CLIA), prior to approval or clearance of a diagnostic device [21]. Liquid biopsy assays may be clinically valuable [10–13] but can be technically challenging. Although there is an increasing use of liquid biopsies in clinical practice, additional clinical validity and utility data is still needed [22]. Many studies assessing the analytical validity of liquid biopsy assays examine concordance between tumor tissue and plasma samples, which introduces confounding variables such as tumor heterogeneity and has the potential to conflate clinical validity with analytical validity [22]. These challenges can be overcome by evaluating analytical validity using samples with known variants at specified variant allele fractions such as cell line DNA diluted in an appropriate matrix [22]. Additionally, an evaluation of the potential impact of preanalytical and analytical variables is crucial [22].

The analyses presented here describe the broad analytical and clinical validation of FoundationOne^®^Liquid CDx (Foundation Medicine, Inc; Cambridge, MA), a novel liquid biopsy CGP platform. The validation of tumor fraction (TF), variant allele fraction (VAF), and other clinical validation studies will be described in detail elsewhere.

## Materials and methods

### Assay methods

FoundationOne Liquid CDx is an FDA-approved next generation sequencing-based in vitro diagnostic device that targets 324 genes utilizing circulating cell-free DNA (cfDNA) isolated from plasma derived from the anti-coagulated peripheral whole blood of cancer patients, performed at Foundation Medicine, Inc (Cambridge, MA) (link to the FDA label [23]). Additional clinical decision insights and genomic analysis are also provided as a professional service under CLIA and College of American Pathologists (CAP) regulations. This assay is the result of the evolution of Foundation Medicine’s FoundationACT and FoundationOne Liquid assays.

**Fig 1:**
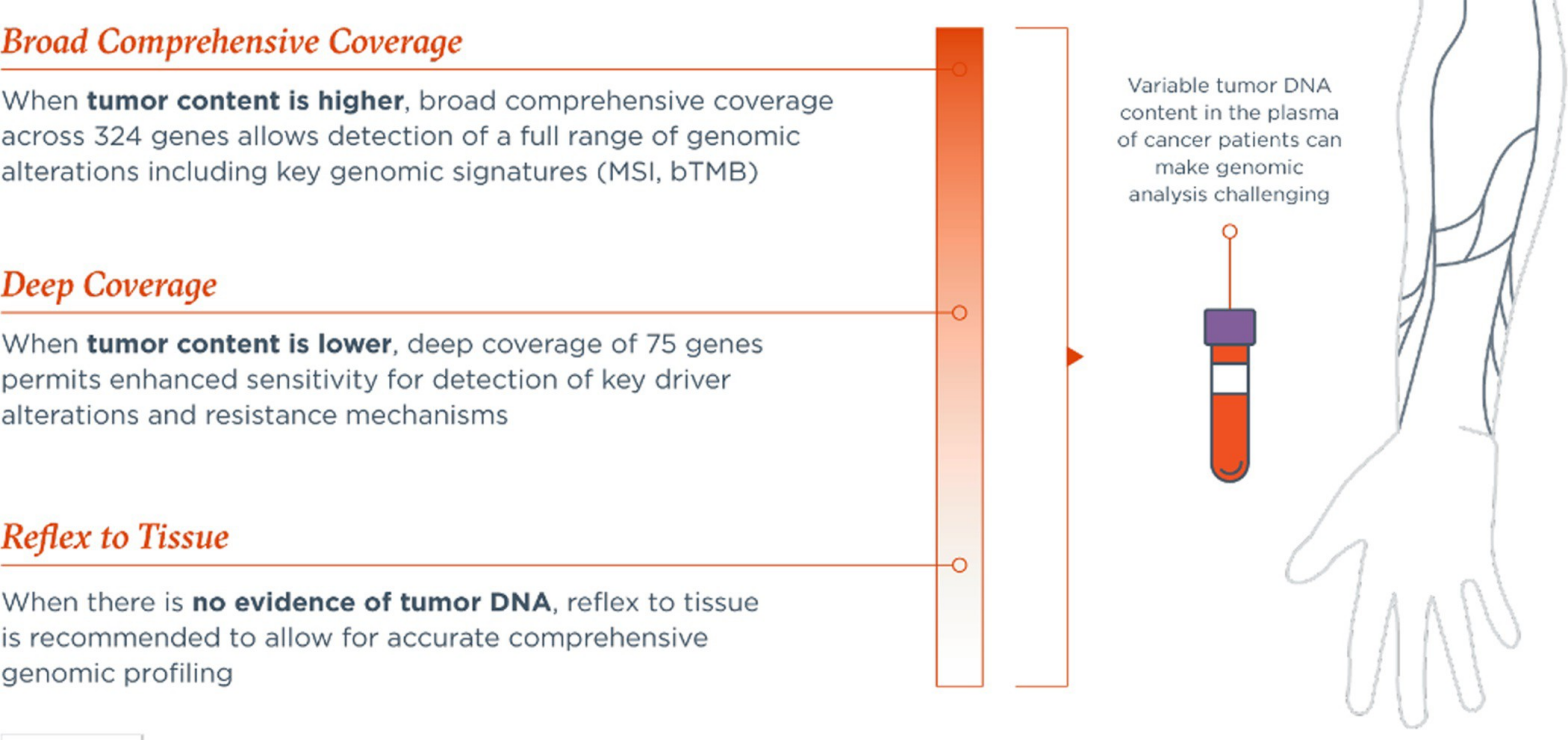
FoundationOne Liquid CDx assay utilization overview. FoundationOne Liquid CDx was designed to allow comprehensive genomic profiling, understanding that shed of tumor DNA can be variable depending on a patient’s clinical characteristics.

All coding exons of 309 genes are targeted; select intronic or non-coding regions are targeted in 21 of these genes. Additionally, select intronic or non-coding regions are targeted in 15 genes, resulting in 324 total targeted genes. Sequence data are processed using a custom analysis pipeline that filters sequencing artifacts and variants known to be benign. Known and likely pathogenic variants implicated in cancer are reported, which may be somatic and/or germline variants. The assay detects substitutions, indels, genomic rearrangements, CNAs (amplifications and losses), and genomic signatures including bTMB, MSI, and TF. Through a novel hybrid capture approach, a subset of targeted regions in 75 genes is baited for greater sensitivity through ultra-deep sequencing coverage (referred to as the enhanced sensitivity region). The enhanced sensitivity region was selected based on genomic regions with increased actionability with current or future targeted therapies (Fig 1). Other targeted genomic regions are baited for high sensitivity through deep sequencing coverage (referred to as the standard sensitivity region). Refer to S1 Table for the complete list of targeted genes.

The FoundationOne Liquid CDx gene content is based on that of the US FDA approved FoundationOne^®^CDx assay. Baited genes and gene regions were chosen based on the current and potential future clinical impact, with the size of the baited region and the ability of the current technology to make confident calls from the baited regions being additional key considerations. Genes with therapeutic, diagnostic, and prognostic relevance, as well as biomarkers that may serve to guide cancer treatment in the future, were included in the assay. In addition, baited regions are included for confident determination of the bTMB and MSI status, complex biomarkers associated with prediction of response to immunotherapy. The FoundationOne Liquid CDx assay is intended to provide genomic information for use by qualified health care professionals in accordance with professional guidelines and is not conclusive or prescriptive for labeled use of any specific therapeutic product unless otherwise noted in the FDA-approved assay labeling.

### Bioinformatics methods

Sequence data is analyzed using mainly proprietary software developed by Foundation Medicine. Reads are demultiplexed (sorted into sets of reads deriving from distinct samples), and their fragment barcodes (FBCs) are extracted and encoded into the read names. For each sample, read pairs with matching FBCs are processed together to: 1) identify clusters of reads originating from the same original fragment, 2) merge overlapping read pairs into single reads, where possible, and 3) generate consensus reads representing all information in the set of reads for each cluster. The consensus reads are then aligned to the reference genome.

For the detection of short variants and rearrangements, a de novo assembly is performed. This is done using proprietary software to generate a de Bruijn graph including all k-mers in reads mapping to a particular locus. For each variant, there is a set of k-mers supporting the variant and a set of k-mers that would support the reference or another variant at the location. Each candidate variant is then scanned against reads in the locus to identify which reads support either the candidate variant or a different variant or reference at the location. The final variant calls are made based on a model that takes into account the coverage at the location, the number of supporting read clusters and their redundancy level, and the number of error-containing clusters.

CNAs are detected using a comparative genomic hybridization-like method. First, a log-ratio profile of the sample is obtained by normalizing the sequence coverage obtained at all exons and genome-wide single-nucleotide polymorphisms (SNPs) against a process-matched normal control. This profile is segmented and interpreted using allele frequencies of sequenced SNPs to estimate tumor purity and copy number at each segment.

To determine MSI status, approximately 2000 repetitive loci (minimum of 5 repeat units of mono-, di-, and trinucleotides) are assessed to determine what repeat lengths are present in the sample. A locus containing a repeat length present in an internal database generated using >3000 clinical samples is considered to be 'unstable'. An MSI indicator is generated by calculating the fraction of unstable loci, considering only those loci that achieve adequate coverage for consideration for the sample. Samples with >0.5% unstable loci are considered to be MSI-High.

Blood tumor mutational burden (bTMB) is measured by counting all synonymous and non-synonymous variants present at 0.5% allele frequency or greater and filtering out potential germline variants according to published databases of known germline polymorphisms including dbSNP and ExAC. Additional germline alterations are assessed for potential germline status and filtered out using a somatic-germline/zygosity algorithm. Furthermore, known and likely driver mutations are filtered out to exclude bias of the data set. The resulting mutation number is then divided by the coding region corresponding to the number of total variants counted, or approximately 750 kilobases (kb). The resulting number is reported in units of mutations per megabase (mut/Mb).

### Sample selection and specimen characteristics

Samples used for assay performance studies consisted of whole blood specimens of cancer patients and cfDNA samples selected from an inventory of residual banked cfDNA isolated from whole blood specimens of cancer patients, representing >30 cancer types (S2 Table). Institutional Review Board approval was obtained from New England IRB prior to use of samples in the described validation studies and all data was anonymized prior to performing the described analyses. Targeted VAFs were achieved by diluting the samples in fragmented buffy coat genomic DNA (gDNA), when required. Assay validation studies were executed between April and December of 2019. cfDNA samples were originally extracted from plasma and frozen as early as May of 2016.

Due to the scarcity of biomarker-positive cfDNA samples, contrived samples were also used. Contrived samples consisted of fragmented cell line DNA diluted in human plasma and titrated to target levels with biomarker-negative cfDNA to mimic a clinical plasma sample. For substitutions, indels, and rearrangements, cell line pools harboring multiple variants were used. Additionally, a plasmid construct was diluted and titrated as described above to represent *NTRK3* rearrangements. In total, >7,500 samples were processed as part of assay validation studies.

### Clinical validation

In addition to analytical validation of the platform, a number of relevant biomarkers were evaluated for clinical validity via clinical bridging, either to a predicate companion diagnostic using clinical samples or to a clinical trial assay using clinical trial samples. A subset of these analyses is presented here. Other clinical validation studies for the assay are in review.

#### Clinical validation for detection of *PIK3CA* alterations

Clinical validity of the assay as an aid in identifying breast cancer patients harboring *PIK3CA* alterations was evaluated through retrospective testing of plasma samples from advanced or metastatic HR-positive, HER2-negative breast cancer patients enrolled in the Novartis clinical trial CBYL719C2301 (SOLAR-1) [24]. The primary endpoint for SOLAR-1 was progression-free survival (PFS) using Response Evaluation Criteria in Solid Tumors (RECIST v1.1), based on investigator assessment. Plasma samples were collected prior to study treatment and analyzed retrospectively for clinical validation. All available samples were considered; sample exclusion criteria included lack of clear identification on stored sample, obvious physical damage of stored sample, and insufficient sample volume. The primary analysis was conducted with eligible samples with the assay’s recommended DNA input and with valid results from both FoundationOne Liquid CDx and the tumor tissue polymerase chain reaction (PCR)-based clinical trial assay (CTA). Of the 572 patients enrolled into the clinical study, 359 were included in the primary analysis. Concordance with the CTA was assessed and PFS based on assay test results was evaluated.

#### Concordance study for *EGFR* exon 19 deletion and *EGFR* exon 21 L858R

Clinical validity of the assay as an aid in identifying patients with advanced NSCLC who may be eligible for treatment with an *EGFR* tyrosine kinase inhibitor (erlotinib, gefitinib, or osimertinib) was established through a non-inferiority study with the FDA-approved cobas EGFR Mutation Test v2 (referred to as the reference assay) following the methods defined in Li et al. (2016) [25]. Samples were prospectively collected from an unrelated clinical trial and were eligible for this analysis if the patients did not enter the clinical study. Samples included in this concordance analysis were selected sequentially starting from a specific testing date until the predefined number of 150 *EGFR*-positive and 100 *EGFR*-negative samples were accrued. One replicate of each sample was tested using FoundationOne Liquid CDx (denoted as CGP) and two replicates were testing using the reference assay (denoted as Ref1 and Ref2). Samples with any missing results were excluded from the analysis. A total of 177 samples were included in this analysis to evaluate the non-inferiority as compared to the reference assay. To show that the agreement (positive percent agreement [PPA] and negative percent agreement [NPA]) between CGP and Ref1/Ref2 is non-inferior to the agreement between Ref1 and Ref2, the estimates of ζPPA1, ζPPA2, ζNPA1 and ζNPA2 and the corresponding one-sided 95% upper bounds confidence limit were computed using the bootstrap method. In which, ζPPA1 is the difference between the PPA of Ref1 and CGP and the PPA of Ref1 and Ref2; ζPPA2 is the difference between the PPA of Ref2 and CGP and the PPA of Ref2 and Ref1; ζNPA1 is the difference between the NPA of Ref1 and CGP and the NPA of Ref1 and Ref2; ζNPA2 is the difference between the NPA of Ref2 and CGP and the NPA of Ref2 and Ref1. The one-sided 95% upper bounds confidence limit of ζPPA1, ζPPA2, ζNPA1 and ζNPA2 were then compared to the pre-defined non-inferiority margin to evaluate non-inferiority as compared to the reference assay for the detection of *EGFR* exon 19 deletions and exon 20 L858R alterations.

### Analytical performance validation

#### Contrived sample functional characterization

To support the use of contrived samples in performance evaluation studies, a contrived sample functional characterization (CSFC) study was performed to demonstrate commutability of test performance when using contrived or clinical specimens. The commutability between clinical and contrived samples was established by testing a dilution series to compare variant detection rates across different alteration types (subs, indels, rearrangements, copy number amplifications, copy number losses, MSI, and bTMB) totaling 924 cfDNA sample replicates and 1069 enzymatically fragmented cell-line gDNA sample replicates (contrived samples).

#### Limit of blank

The limit of blank (LoB) describes the highest measurement result that is likely to be observed for a blank sample with a stated probability α [27]. According to industry standard, an α (type I error rate, false positive rate) of 0.05 was selected. The LoB was established by profiling 30 cfDNA samples from asymptomatic individuals without cancer with 4 replicates per sample (>130,000 variants evaluated). Donors were all over the age of 60 and included smokers and non-smokers with the intent of representing an increased occurrence of clonal hematopoiesis. The LoB was estimated via the non-parametric method.

#### Limit of detection

The limit of detection (LoD) describes the lowest level at which an analyte (genomic variant) can be consistently detected [26]. According to industry standard, consistently detected was defined the level at which a 95% detection rate is observed. The LoD for each variant type was established by processing a total of 1069 tests across 10 contrived samples representing short variants, rearrangements, CNAs, bTMB component variants, and MSI. The LoD was defined as the lowest dilution level tested with ≥95% detection across replicates. For variants with observed hit rates between 10% and 90% for 3 levels, the probit model was used to determine LoD. The LoD estimates were determined as either VAF for subs, indels, rearrangements, and bTMB component variants; TF for CNAs; or percent unstable loci for MSI. Short variants with hit rates of ≥95% at all dilution levels or hit rates <95% for all dilution levels were excluded from analysis as LoD could not be reliably estimated. As bTMB score is an index variable in which qualifying substitutions and indels are counted and the resulting score normalized across the genomic region over which the score is calculated, the LoD of the component variants were determined in the evaluation of bTMB LoD. A subset of clinically actionable alterations were selected for analysis based on currently available targeted therapies and therapies currently under evaluation.

#### Precision: Reproducibility and repeatability

We evaluated the reproducibility and repeatability (precision) of the assay for tumor profiling variants (platform-wide analysis), a subset of select clinically actionable alterations, MSI, and bTMB. Repeatability (intra-run: replicates processed on the same plate under the same conditions) and reproducibility (inter-run: replicates processed on different plates under different conditions) were assessed across 3 reagent lots, 2 sequencers, and 2 processing runs, with 2 replicates per run (24 replicates per sample). Confidence was calculated using two-sided exact 95% confidence intervals (CI). For the tumor profiling variants and the subset of select clinically actionable alterations, all 47 samples were used to evaluate assay precision, including 16 contrived samples and 31 clinical cfDNA samples. Precision of reporting of MSI status was evaluated across all 47 samples included in this study. Precision of bTMB scores was evaluated across samples with bTMB scores ≥5 muts/Mb.

#### Analytical accuracy

Short variant and rearrangement detection rates were compared to that of an externally validated cfDNA-based NGS assay. A total of 282 samples (272 cfDNA and 10 contrived) representing 37 tumor types were tested and variant detection was compared in the 74 genes common to both assays. Clinical samples were selected from archival cfDNA samples from clinical testing originally processed as early as May 2016 while contrived samples using the methods described above were used to represent rare alterations. Concordance was assessed for short variants and rearrangements across the 74 genes common to both platforms. Concordance was also assessed more specifically for a subset of clinically actionable alterations. In a separate analysis, the detection of *PIK3CA* alterations were compared to another orthogonal cfDNA-based NGS method using residual plasma samples from the Novartis clinical trial CBYL719C2301 (SOLAR-1) [24].

## Results

### Clinical validation

#### Clinical validation for detection of *PIK3CA* alterations

Of 572 patients enrolled in SOLAR-1, 432 had available baseline plasma samples. The characteristics of the patients at baseline have been described previously [24]. Results were available for 375 patients for inclusion in the primary analysis and considered for the concordance analysis summarized in Table 1. A PPA of 71.7% and an NPA of 100% for the detection of eligible *PIK3CA* alterations were observed as compared to the tumor tissue PCR CTA. The PPA observed for detection of *PIK3A* alterations was likely impacted by the use of banked clinical trial samples, as many samples were tested with plasma volumes that were significantly lower than those that would be expected from the recommended assay input of 17 mL of whole blood. Additionally, variability in the shed rate of tumor DNA into the bloodstream [13] could also contribute to reduced detection rate in cfDNA.

**Table 1:** Concordance between FoundationOne Liquid CDx and alpelisib CTA^a^ for eligible *PIK3CA* alterations^b^.

	CTA Positive	CTA Negative	Invalid	Total
**cfDNA CGP Positive**	165	0	1	166
**cfDNA CGP Negative**	65	129	3	197
**Invalid**	7	5	0	12
**Total**	237	134	4	375
	PPA (95% CI): 71.7% (65.4%, 77.5%)	NPA (95% CI): 100% (97.2%, 100%)		

^a^Tumor tissue PCR assay

^b^Defined as alterations with the amino acid effect: C420R, E542K, E545A, E545D (1635G>T only), E545G, E545K, Q546E, Q546R, H1047L, H1047R, H1047Y.

cfDNA = cell-free DNA; CI = confidence interval; CTA = clinical trial assay; NPA = negative percent agreement; PPA = positive percent agreement.

A total of 16 samples from the primary analysis set had invalid results from one or both assays. The evaluable population encompassed 230 *PIK3CA* alteration-positive patients and 129 *PIK3CA*-negative patients. Comparable demographics and baseline clinical characteristics were demonstrated for evaluable and unevaluable patient populations in the primary analysis set for both *PIK3CA*-positive and *PIK3CA*-negative patients. The primary analysis set was also shown to be representative of the overall SOLAR-1 patient population (not shown).

Alpelisib in combination with fulvestrant was evaluated in the plasma-positive population (n = 165) with an estimated 54% risk reduction in disease progression or death in the alpelisib plus fulvestrant arm compared to the placebo plus fulvestrant arm (hazard ratio[HR]: 0.46, 95% CI: 0.30, 0.70). Median PFS was 11.0 months for the alpelisib plus fulvestrant arm versus 3.6 months for the placebo plus fulvestrant arm (Table 2).

**Table 2:** Progression-free survival among 165 CTA-positive/ FoundationOne Liquid CDx-positive patients.

PFS	Alpelisib + Fulvestrant n = 84	Placebo + Fulvestrant n = 81
**No of events (%)**	54 (64.3)	67 (82.7)
**PD (%)**	52 (61.9)	61 (75.3)
**Death (%)**	2 (2.4)	6 (7.4)
**No of censored (%)**	30 (35.7)	14 (17.3)
**Months, median (95% CI)**^a^	11.0 (7.3, 15.9)	3.6 (2.4, 5.8)
**HR**^b^ **(95% CI) Alpelisib + Fulvestrant /Placebo + Fulvestrant**^**1**^	0.46 (0.30, 0.70)	

^a^The 95% CI calculated from PROC LIFETEST output using the method of Brookmeyer and Crowley (1982).

^b^HR estimated using Cox regression model stratified by the 2 stratification factors: presence of lung and/or liver metastases, previous treatment with any CDK4/6 inhibitor, and adjusted for clinically relevant covariates, as well as the imbalanced covariates.

CI = confidence interval; CTA = clinical trial assay; HR = hazard ratio; PD = progressive disease; PFS = progression-free survival.

#### Concordance study for *EGFR* exon 19 deletions and *EGFR* exon 21 L858R alterations

A total of 177 samples from NSCLC patients with two valid replicate results by the reference assay (denoted as Ref1 and Ref2) and one replicate by FoundationOne Liquid CDx (denoted as CGP) were included in this analysis. The concordance data are summarized in Tables 3 and 4 and the non-inferiority comparison is provided in Table 5.

**Table 3:** Concordance between FoundationOne Liquid CDx and cobas EGFR Mutation Test v2 for *EGFR* exon 19 deletions and exon 21 L858R alterations.

	Ref1 Positive			Ref1 Negative		
	Ref2 Positive	Ref2 Negative	Total	Ref2 Positive	Ref2 Negative	Total
**cfDNA CGP Positive**	**80**	**4**	**84**	**1**	**3**	**4**
**cfDNA CGP Negative**	**2**	**0**	**2**	**0**	**87**	**87**
**Total**	**82**	**4**	**86**	**1**	**90**	**91**

cfDNA = cell-free DNA; CGP = FoundationOne Liquid CDx; Ref1 = reference assay replicate 1; Ref2 = reference assay replicate 2.

**Table 4:** Non-inferiority concordance study for clinical validity for the detection of *EGFR* exon 19 deletions and L858R substitutions in NSCLC (n = 177).

Comparison	PPA	NPA
**Ref2|Ref1**	**95.3%**	**98.9%**
**Ref1|Ref2**	**96.1%**	**98.7%**
**CGP|Ref1**	**97.7%**	**95.6%**
**CGP|Ref2**	**97.7%**	**95.4%**

CGP = FoundationOne Liquid CDx; NPA = negative percent agreement; PPA = positive percent agreement; Ref1 = reference assay replicate 1; Ref2 = reference assay replicate 2.

**Table 5:** Point estimate and one-sided 95% upper confidence limit of ζPPA1, ζNPA1, ζPPA2, and ζNPA2.

	Point Estimate	Mean one-sided 95% upper confidence limit
ζ_PPA1_	-2.3%	2.3%
ζ_NPA1_	3.3%	6.6%
ζ_PPA2_	-1.6%	4.7%
ζ_NPA2_	3.3%	6.6%

NPA = negative percent agreement; PPA = positive percent agreement.

This study establishes the clinical validity of the assay and the non-inferiority to plasma testing with cobas EGFR Mutation Test v2 for the identification of patients eligible for treatment with erlotinib, gefitinib, and osimertinib.

### Analytical performance validation

#### Contrived sample functional characterization

The hit rates for contrived samples and cfDNA samples were evaluated and compared across targeted variant concentrations. The hit rate was consistent across contrived and clinical cfDNA samples (S3 Table). The cfDNA sample and contrived sample targeted levels at which ≥95% hit rate was observed, respectively were: 0.30% and 0.35% VAF for short variants; 0.20% and 0.30% VAF for rearrangements; 5% and 5% tumor fraction for copy number alterations; 1% and 0.8% unstable loci for MSI; 1.5% and 1.0% VAF for bTMB component indels; 1.1% and 1.0% VAF for bTMB component substitutions. The comparable hit rates between clinical and contrived samples at matching targeted dilution levels supports the use of contrived samples to establish assay performance.

#### Limit of blank

Of the 120 sample replicates tested, 79 generated valid results for inclusion in the LoB study. As asymptomatic donors were used for this study, an increased sample insufficiency rate was observed, partly due to a decreased cfDNA concentration in the blood of these donors as compared to cancer patients. Across 79 replicates, 1,735 unique variants were included in the analysis for a total of 137,065 data points. A total of 18 positive calls were observed across 4 unique short variants. The LoB was determined to be the ideal value of zero for short variants, rearrangements and CNAs. The detection rate in these healthy donors was shown to be 0% for rearrangements and CNAs and 0.013% (~1 in 8000) for short variants (substitutions and indels). Cancer-related variants detected in these samples were observed in all replicates indicating that these may be true somatic alterations harbored by the otherwise healthy donors.

#### Limit of detection

**Fig 2:**
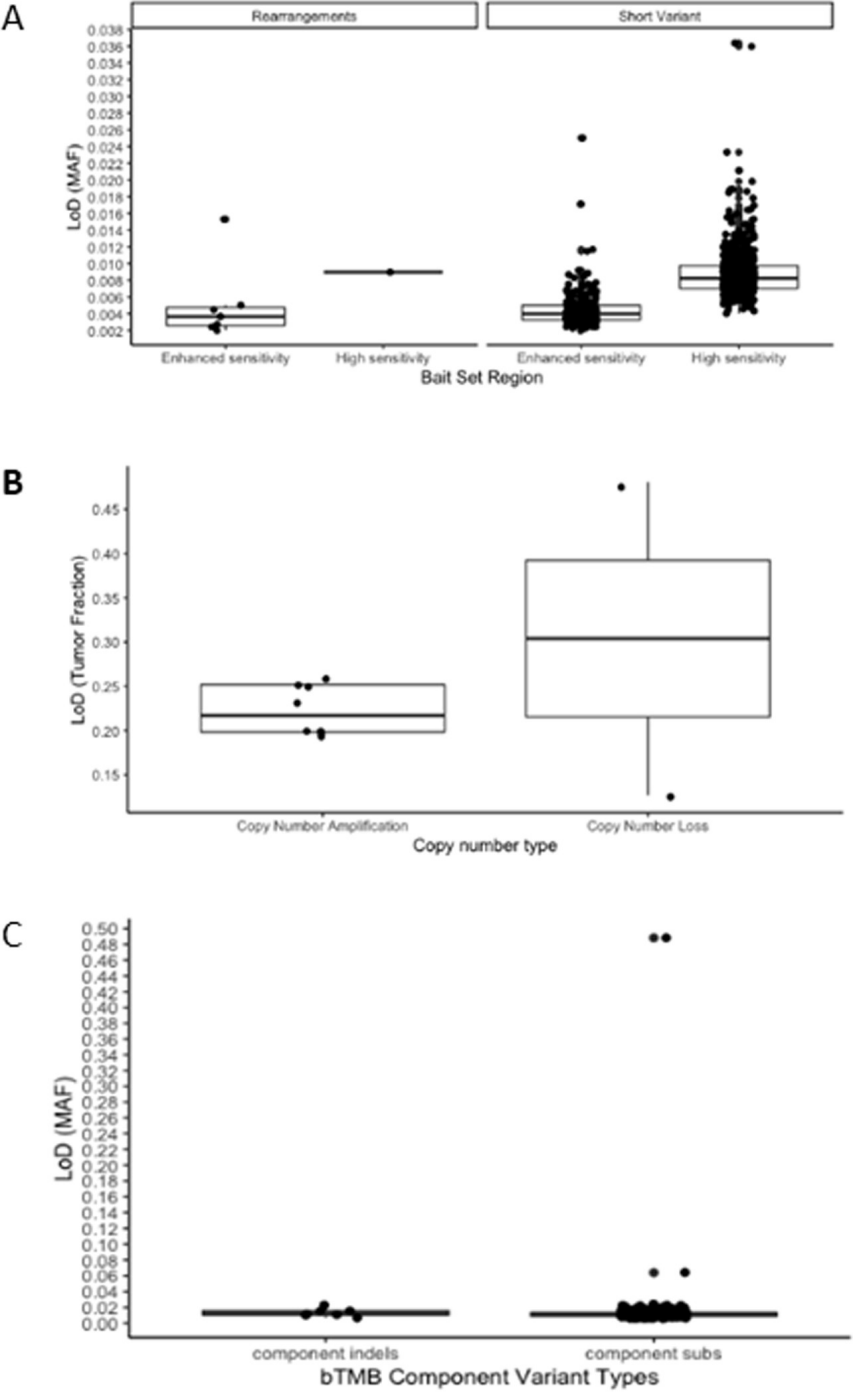
Box blots representing limits of detection (LoD) for variant categories in which 1069 tests were performed and 2180 variants were analyzed. A. Short variant and rearrangement LoD, B. Copy number LoD, C. bTMB component LoD. MAF–Mutant Allele Fraction.

The LoD data for each variant category is presented in Fig 2. A total of 864 short variants were included in the LoD analysis: 269 in the enhanced sensitivity region and 595 in the standard sensitivity region of the bait set. The LoD for short variants was 0.40% VAF for the enhanced sensitivity region and 0.82% VAF for the standard sensitivity region of the bait set. Eight rearrangements were evaluated, resulting in an LoD for rearrangements of 0.37% VAF in the enhanced sensitivity region and 0.90% in the standard sensitivity region.

A total of 8 unique copy number amplification variants ranging from 7 to 26 copies were included in the LoD analysis with hit rates of ≥95% across all dilution levels observed for 4 of the copy number amplifications analyzed, signifying that the true LoD is lower than 19.8% TF, the lowest level tested in this study. The median copy number amplification LoD determined in this study was 22% TF. The mean LoD for copy number losses, determined across 2 genes, was 30.4% TF. The LoD for MSI was 0.8% unstable loci. The LoD for bTMB was 1.00% for both component indels and substitutions.

The median LoD for a subset of clinically actionable alterations (Table 6) were consistent with the LoD determined for the corresponding variant category (Fig 2), confirming the applicability of the variant category analysis across the genomic regions targeted by the assay.

**Table 6:** Limits of detection of a subset of clinically-actionable alterations in which 940 tests were performed and 46 unique variants were analyzed.

Gene	Alteration Sub-Type	Median LoD
*ATM*	Indels	0.51% VAF
	*ATM-EXPH5* Truncation^a^	1.13% VAF
*BRAF*	Substitutions	0.33% VAF
*BRCA1*	Indels	0.38% VAF
	Substitutions	0.34% VAF
	Rearrangement^a^	0.87% VAF
*BRCA2*	Indels	0.36% VAF
	*BRCA2- EDA* Truncation^a^	0.48% VAF
	Copy Number Loss	48.1% TF
*EGFR*	Indels (exon 19 deletions)	0.27% VAF
	Substitutions (L858R substitutions)	0.34% VAF
*KRAS*	Substitutions	0.33% VAF
*MET*	Indels	0.41% VAF
*NRAS*	Substitutions	0.42% VAF
*PALB2*	Indels	0.37% VAF
	Substitutions	0.51% VAF
*PIK3CA*	Substitutions	0.34% VAF
*ALK*	*ALK-EML4* Rearrangement	0.24% VAF
	*NPM1-ALK* Rearrangement	0.94% VAF
*NRTK1*	*NTRK1-TPM3* Rearrangement	0.44% VAF
*NTRK3*	*NTRK3-ETV6* Rearrangement	0.27% VAF
*RET*	*RET-CCDC6* Rearrangement	0.20% VAF
*ROS1*	*ROS1-GOPC* Rearrangement	0.75% VAF
	*ROS1-SLC34A2* Rearrangement	0.28% VAF
*ERBB2*	Copy Number Amplification	19.8% TF
*PTEN*	Copy Number Loss	12.7% TF

TF = tumor fraction; VAF = variant allele fraction.

Additionally, the LoD was confirmed for some rare alteration types using clinical cfDNA specimens tested across 24 replicates using a precision study design targeting approximately 1.5x LoD, as described above in the Precision section. Because a major component driving the detectability of a variant is genomic context (repetitiveness of the reference genomic region), the LoD analysis for short variants was also evaluated within categories based on genomic context as summarized in S4 Table.

#### Precision: Reproducibility and repeatability

A total of 1240 individual variants were evaluated for the platform-wide precision analysis. A total of 691,920 variant pairs were evaluated for repeatability and 1,390,040 variant data points were evaluated for reproducibility. In this precision study, a success rate of 99.38% (1121 of 1128 tests) was observed. The overall repeatability for all variants was 99.47% (95% CI: 99.45%, 99.48%). The overall reproducibility for all variants was 99.59% (95% CI: 99.58%, 99.60%). Similar to the LoD analysis, reproducibility was evaluated within variant categories based on genomic context. The reproducibility result for each variant type are summarized in Table 7.

**Table 7:** Reproducibility of platform-wide variant detection among 47 samples.

n = 1240 unique variants n = 1,390,040 data points			
Variant Type	Unique Variants	Concordant Data Points	Reproducibility (95% CI)
Substitutions	898	1,002,981 of 1,006,658	99.63% (99.62%, 99.65%)
Substitution in a non-repetitive region or a repetitive region of ≤7 base pairs	882	985,150 of 988,722	99.64% (99.63%, 99.65%)
Substitution in a repetitive region of >7 base pairs	16	17,831 of 17,936	99.41% (99.29%, 99.52%)
Indels	228	254,509 of 255,588	99.58% (99.55%, 99.60%)
Indel in non-repetitive region or a repetitive region of ≤3 base pairs	52	58,054 of 58,292	99.59% (99.54%, 99.64%)
Indel in a repetitive region of 4 to 6 base pairs	118	131,816 of 132,278	99.65% (99.62%, 99.68%)
Indel in a repetitive region of ≥7 base pairs	58	64,639 of 65,018	99.42% (99.36%, 99.47%)
Rearrangements	60	66,723 of 67,260	99.20% (99.13%, 99.27%)
Copy Number Alterations	54	60,115 of 60,534	99.31% (99.24%, 99.37%)
Copy Number Amplification (4–5 copies)	1	1,121 of 1,121	100.00% (99.67%, 100.00%)
Copy Number Amplification (>6 copies)	48	53,402 of 53,808	99.25% (99.17%, 99.32%)
Copy Number Loss	5	5,592 of 5,605	99.77% (99.60%, 99.88%)
Total	1240	1,384,328 of 1,390,040	99.59% (99.58%, 99.60%)

CI = confidence interval

For the subset of select clinically actionable alterations, 47 samples (16 contrived samples and 31 clinical cfDNA samples) were evaluated across 24 replicates for a total of 1128 tests. Repeatability of 100% was observed for 43 alterations and ≥90% repeatability was observed for 53 alterations. For the 57 targeted variants assessed, the overall repeatability was 96.39% (95% CI: 95.28%, 97.30%). Reproducibility of 100% was observed for 42 alterations and ≥90% reproducibility was observed for 55 alterations. For the 57 targeted variants assessed, the overall reproducibility was 97.33% (95% CI: 96.67%, 97.89%). Reproducibility results are presented in S5 Table.

Repeatability and reproducibility of MSI status was 100% for 46 samples. For one sample, a single discordant call was observed. For the evaluation of bTMB, for repeatability the % coefficient of variation (CV) ranged from 5.9% to 13.5% and for reproducibility the %CV ranged from 6.0% to 16.4 (S6 Table).

Twenty-nine cfDNA samples with variants near LoD were evaluated to confirm LoD and precision in clinical specimens. Twenty-six samples had 100% reproducibility, one had 95.8% reproducibility, one had 87.5% reproducibility, and one had 91.67% reproducibility (S7 Table).

#### Analytical accuracy

A total of 282 samples (272 cfDNA and 10 contrived) were analyzed. Using a cfDNA-based NGS assay as the reference, a short variant PPA of 96.2% (95% CI: 94.8%, 97.4%) and an NPA (95% CI) of >99.9% (99.9%, 100.0%) were observed. For rearrangement detection, PPA (95% CI) was 100.0% (59.04%, 100.0%) and NPA (95% CI) was 99.8%, (99.5%, 100.0%) (Table 8). A PPA of 100% was observed for a subset of select clinically actionable alterations, demonstrating the analytical accuracy of the assay (Table 8). A scarcity of samples with sufficient cfDNA for both assays for some alterations led to a small sample size for some of the targeted variants in Table 8. Analytical accuracy data from a subset of clinically actionable alterations is summarized in Table 9.

**Table 8:** Concordance of FoundationOne Liquid CDx and an externally validated cfDNA NGS assay for platform-wide variants (n = 912 positive variants; n = 157,008 negative variants as determined by the comparator assay).

Variant Type	CDx(+) Ref(+)	CDx(-) Ref(+)	CDx(+) Ref(-)	CDx(-) Ref(-)	PPA (95% CI)	NPA (95% CI)	OPA (95% CI)
All Short Variants (Substitutions and Indels)	871	34	8	155315	96.2% (94.8%, 97.4%)	>99.9% (99.9%, 100.0%)	>99.9% (99.9%, 100.0%)
Substitutions	848	34	8	151954	96.2% (94.7%, 97.3%)	>99.9% (99.9%, 100.0%)	>99.9% (99.9%, 100.0%)
Indels	23	0	0	3361	100.0% (85.18%, 100.0%)	100.0% (99.89%, 100.0%)	100.0% (99.89%, 100.0%)
Rearrangements	7	0	3	1682	100.0% (59.04%, 100.0%)	99.8% (99.5%, 100.0%)	99.8% (99.5%, 100.0%)

CDx = FoundationOne Liquid CDx; cfDNA = cell-free DNA; CI = confidence interval; NPA = negative percent agreement; PPA = positive percent agreement; Ref = reference assay.

**Table 9:** Concordance of FoundationOne Liquid CDx and an externally validated cfDNA NGS assay for select clinically actionable alterations (a subset of the results in Table 8).

Alteration	n	PPA (95% CI)	NPA (95% CI)
*EGFR* L858R	10	100% (69.2%, 100.0%)	100% (98.7%, 100.0%)
*EGFR* Exon 19 non-frameshift deletions	11	100% (71.5%, 100.0%)	100% (99.7%, 100.0%)
*PIK3CA* base substitutions	49	100% (92.7%, 100.0%)	100% (99.9%, 100.0%)
*ALK* rearrangements	1	100% (2.5%, 100.0%)	99.9% (99.7%, 100.0%)
*NTRK1* rearrangements	3	100% (29.2%, 100.0%)	100% (99.8%, 100.0%)
*ROS1* rearrangements	1	100% (2.5%, 100.0%)	100% (99.8%, 100.0%)
*BRCA1* short variants	1	100% (2.5%, 100.0%)	100% (98.7%, 100.0%)
*BRCA2* short variants	2	100% (15.8%, 100.0%)	100% (99.3%, 100.0%)

cfDNA = cell-free DNA; CI = confidence interval; NGS = next-generation sequencing; NPA = negative percent agreement; PPA = positive percent agreement; Ref = reference assay

In the concordance analysis of *PIK3CA* alterations detection as compared to a second cfDNA-based NGS method, 412 samples were evaluable by both assays. Concordance was evaluated using the 412 samples that generated results using both platforms. The overall percent agreement (OPA) (95% CI) was 93.93% (91.17, 63.04). See S8 Table for detailed analyses.

## Discussion

Tumor tissue-based testing has historically been the standard of care for genomic analysis, but in many situations adequate tissue sampling is not possible, thus the role of liquid biopsy has rapidly evolved as another option for initial genomic testing. Liquid biopsy also offers opportunities to explore the underlying evolving tumor in a minimally invasive way to help inform cancer management. Additionally, due to intratumor heterogeneity, a tumor biopsy may represent a small sample of the overall tumor cell population, a limitation that can potentially be overcome with liquid biopsy [10, 16–18]. While, it has been recognized that patients could benefit from a cfDNA-based CGP assay (liquid biopsy) with proven performance [13], concern has been expressed around the robustness and lack of performance data available for cfDNA-based genomic tests [27].

The FoundationOne Liquid CDx panel is based on the gene content of the FoundationOne^®^CDx assay, the first FDA-approved tissue-based broad companion diagnostic that is analytically and clinically validated for all solid tumors. The ability of the FoundationOne Liquid CDx assay to also robustly detect genomic alterations demonstrated in tissue to predict response to targeted therapies has been demonstrated in this study.

The analytical performance of FoundationOne Liquid CDx was evaluated across >7,500 tests and >30,000 unique variants over >300 genes and >30 cancer types, allowing for a comprehensive assessment of performance. Across >900 variants the high sensitivity of the assay was demonstrated with a median 95% LoD of 0.40% VAF in the enhanced sensitivity region and 0.82% VAF in the standard sensitivity region for short variants; 0.37% VAF and 0.90% VAF in the enhanced and standard sensitivity regions, respectively, for rearrangements; 21.7% TF for copy number amplifications; and 30.4% TF for copy number losses. Through the analysis of >130,000 variants, the variant detection rate in healthy donors was determined to be 0% for rearrangements and CNAs and 0.013% (~1 in 8000) for short variants (substitutions and indels). The high reproducibility of variant calling was demonstrated with 99.59% reproducibility across more than 1 million data points. Across >900 positive variants and >150,000 negative variants, an overall PPA of 96.3% and NPA of >99.9% was observed when comparing to an orthogonal cfDNA-based NGS method. Together, these data demonstrate robust genomic analysis results from the test. The analytic performance demonstrated for the FoundationOne Liquid CDx assay is comparable to other NGS-based broad molecular profiling liquid biopsy assays [28–30].

The body of evidence of regarding correlation between liquid biopsy results and patient response to therapy is growing [31–36]. For example, the clinical utility for the selection of patients for therapy with alectinib using an early version of the Foundation Medicine liquid biopsy assay has also been demonstrated in a prospective clinical study described in the Blood First Assay Screening Trial (BFAST) [31].

Critically, clinical validity, particularly through bridging studies, provides confidence in genomic profiling results for cancer patients. In this study, we also describe the clinical validation of the FoundationOne Liquid CDx assay, including large-scale comparisons with orthogonal clinical plasma- and tissue-genotyping methods, for both *EGFR* in NSCLC and *PIK3CA* in breast cancer. In 2016, the Roche cobas *EGFR* Mutation Test v.2 became the first US FDA approved cfDNA companion diagnostic for patients with NSCLC. The performance comparison between FoundationOne Liquid CDx assay and this orthogonal assay for detection of *EGFR* exon 19 deletions and exon 21 L858R alterations demonstrated non-inferiority concordance.

The clinical bridging study for the second US FDA approved liquid biopsy-based companion diagnostic test demonstrated a 55% PPA (95% CI 49.0, 60.1) and 97% NPA (95% CI 94.0, 99.0) between tissue and liquid results [37]. A similar performance comparison approach between tissue and liquid was utilized in the FoundationOne Liquid CDx validation studies. The clinical validity of the FoundationOne Liquid CDx assay to identify breast cancer patients harboring *PIK3CA* alterations eligible for treatment with alpelisib was assessed through retrospective testing of plasma samples. In this bridging study, all available plasma samples from patients collected at baseline prior to randomization into the SOLAR-1 clinical trial [24] were tested with FoundationOne Liquid CDx, with results compared to tissue genotyping performing using the SOLAR-1 CTA. The PPA and NPA between FoundationOne Liquid CDx and the tissue-based CTA assay were 71.7% (95% CI 65.4%, 77.5%) and 100% (97.2%, 100%), respectively. Most notably, the clinical efficacy of alpelisib in combination with fulvestrant for the FoundationOne Liquid CDx-positive population was demonstrated with an estimated 54% risk reduction in disease progression or death in the alpelisib plus fulvestrant arm compared to the placebo plus fulvestrant arm (HR = 0.46, 95% CI: 0.30, 0.70).

The number of targeted therapies and actionable alterations continues to grow in many tumor types, including breast cancer [13]. While *PIK3CA* alterations occur in 36% or more of breast cancer patients [38] there are other relevant biomarkers to consider in these patients, including *ERBB2*, *BRCA1*, *BRCA2*, *NTRK*, and MSI (or mismatch repair deficiency) [13]. Furthermore, targetable alterations ae increasingly being identified across numerous other solid tumor types, including non-small cell lung cancer [39], prostate cancer [40], colorectal and other gastrointestinal malignancies [41], ovarian cancer [42], melanoma [43], and others. The studies herein describe the ability to interrogate and identify alterations in these relevant genes in a single assay, providing pertinent genomic information from a single, minimally invasive molecular test.

Throughout an individual cancer patient’s journey, liquid and/or tissue testing may be most appropriate to identify genomic alterations indicative of response or resistance to therapy. While the utilization of a robustly validated liquid biopsy assay has definite advantages, including minimally invasive blood sampling and providing a more comprehensive representation of the patient’s entire tumor burden, the variable extent of tumor shedding into the plasma and resultant ctDNA levels can make genomic analysis challenging. Thus, the availability of well-aligned CGP-based tumor- and liquid-based testing assays can maximize benefits to patients. For patients with a high ctDNA content, broad comprehensive coverage across the targeted 324 gene FoundationOne Liquid CDx panel allows detection of a full range of genomic alterations, including key genomic signatures, including MSI and bTMB. For those patients where ctDNA content is lower, deep coverage of 75 genes permits enhanced sensitivity for the detection of key driver alterations and resistance mechanisms. In cases where no ctDNA is detected, reflex to tissue testing is recommended, if possible, to allow for accurate comprehensive genomic profiling to enable data-driven treatment decisions.

The results of the extensive studies presented here demonstrate that FoundationOne Liquid CDx accurately and reproducibly detects the major types of genomic alterations as well as complex biomarkers, such as MSI, bTMB, and tumor fraction. The data described here support the validity and utility of using a well-validated cfDNA-based CGP assay in the therapeutic management of cancer patients. The comprehensive nature of the described performance evaluation studies demonstrates the reliability of test results which can provide confidence to physicians in the use of this assay to provide genomic profiling results for their patients.

## Supporting information

S1 Table The assay interrogates 324 genes, including 309 genes with complete exonic (coding) coverage and 15 genes with only select non-coding coverage (indicated with an *); 75 genes (indicated in bold) are captured with increased sensitivity and have complete exonic (coding) coverage unless otherwise noted. (DOCX)

S2 Table 37 cancer types represented in validation studies. (DOCX)

S3 Table Hit rate comparison between contrived and clinical specimens. (DOCX)

S4 Table Contrived samples platform LoD. (DOCX)

S5 Table Reproducibility study results for a subset of clinically-actionable variants. (DOCX)

S6 Table Precision of bTMB. (DOCX)

S7 Table Confirmation of LoD and precision in cfDNA specimens. (DOCX)

S8 Table Comparison of FoundationOne Liquid CDx with the reference assay for the detection of *PIK3CA* alterations. (DOCX)

S1 File (ZIP)
