# Supplementary material for: Clinical and analytical validation of FoundationOne Liquid CDx, a novel 324-Gene cfDNA-based comprehensive genomic profiling assay for cancers of solid tumor origin
Source: PLoS One. 2020 Sep 25;15(9):e0237802. doi: 10.1371/journal.pone.0237802 (PMC7518588; doi:10.1371/journal.pone.0237802)
Supplement: S1 Table — (DOCX) [file pone.0237802.s001.docx]

S1 Table. The assay interrogates 324 genes, including 309 genes with complete exonic (coding) coverage and 15 genes with only select non-coding coverage (indicated with an *); 75 genes (indicated in bold) are captured with increased sensitivity and have complete exonic (coding) coverage unless otherwise noted.

| ***ABL1 [Exons 4-9]*** | *CASP8* | ***DDR2 [Exons 5,17,18]*** | *FGFR4* | *KDR* | ***MYD88 [Exon 4]*** | *PPP2R2A* | ***SMO*** |
| --- | --- | --- | --- | --- | --- | --- | --- |
| *ACVR1B* | *CBFB* | *DIS3* | *FH* | *KEAP1* | *NBN* | *PRDM1* | *SNCAIP* |
| ***AKT1 [Exon 3]*** | *CBL* | *DNMT3A* | *FLCN* | *KEL* | ***NF1*** | *PRKAR1A* | *SOCS1* |
| *AKT2* | ***CCND1*** | *DOT1L* | *FLT1* | ***KIT [Exons 8,9,11,12,13,17]*** | *NF2* | *PRKCI* | *SOX2* |
| *AKT3* | *CCND2* | *EED* | ***FLT3 [Exons 14,15,20]*** | *KLHL6* | *NFE2L2* | *PTCH1* | *SOX9* |
| ***ALK [Exons 20-29,  Introns 18,19]*** | *CCND3* | ***EGFR [Introns 7,****15****,24-27]*** | ***FOXL2*** | *KMT2A  (MLL)* | *NFKBIA* | ***PTEN*** | *SPEN* |
| *ALOX12B* | *CCNE1* | *EP300* | *FUBP1* | *KMT2D  (MLL2)* | *NKX2-1* | ***PTPN11*** | *SPOP* |
| *AMER1* | *CD22* | *EPHA3* | *GABRA6* | ***KRAS*** | *NOTCH1* | *PTPRO* | *SRC* |
| ***APC*** | *CD70* | *EPHB1* | *GATA3* | *LTK* | *NOTCH2* *[Intron 26]* | *QKI* | *STAG2* |
| ***AR*** | *CD74* {Introns 8-6}* | *EPHB4* | *GATA4* | *LYN* | *NOTCH3* | *RAC1* | *STAT3* |
| ***ARAF [Exons 4,5,7,11,13,15,16]*** | *CD79A* | ***ERBB2*** | *GATA6* | *VAF* | ***NPM1 [Exons 4-6,8,10]*** | *RAD21* | ***STK11*** |
| *ARFRP1* | *CD79B* | ***ERBB3 [Exons 3,6,7,8,10,12,20,21,23,24,25]*** | *GID4  (C17orf39)* | ***MAP2K1 [Exons 2,3]*** | ***NRAS [Exons 2,3]*** | *RAD51* | *SUFU* |
| *ARID1A* | ***CD274*** | *ERBB4* | ***GNA11 [Exons 4,5]*** | ***MAP2K2 [Exons 2-4,6,7]*** | *NT5C2* | *RAD51B* | *SYK* |
| *ASXL1* | *CDC73* | *ERCC4* | *GNA13* | *MAP2K4* | ***NTRK1 [Exons 14,15, Introns 8-11]*** | *RAD51C* | *TBX3* |
| ***ATM*** | ***CDH1*** | *ERG* | ***GNAQ [Exons 4,5]*** | *MAP3K1* | *NTRK2* *[Intron 12]* | *RAD51D* | *TEK* |
| ***ATR*** | ***CDK12*** | ***ERRFI1*** | ***GNAS [Exons 1,8]*** | *MAP3K13* | ***NTRK3 [Exons 16,17]*** | *RAD52* | *TET2* |
| *ATRX* | ***CDK4*** | ***ESR1 [Exons 4-8]*** | *GRM3* | *MAPK1* | *NUTM1* {Intron 1}* | *RAD54L* | *TERC* {ncRNA}* |
| *AURKA* | ***CDK6*** | *ETV4* {Intron 8}* | *GSK3B* | *MCL1* | *P2RY8* | ***RAF1 [Exons 3,4,6,7,10,14,15,17]*** | ***TERT* {Promoter}*** |
| *AURKB* | *CDK8* | *ETV5* {Introns 6, 7}* | *H3F3A* | ***MDM2*** | ***PALB2*** | *RARA* | *TGFBR2* |
| *AXIN1* | *CDKN1A* | ***ETV6* {Introns 5,6}*** | *HDAC1* | *MDM4* | *PARK2* | ***RB1*** | *TIPARP* |
| *AXL* | *CDKN1B* | *EWSR1* {Introns 7, 13}* | *HGF* | *MED12* | *PARP1* | *RBM10* | *TMPRSS2* {Introns 1-3}* |
| *BAP1* | ***CDKN2A*** | ***EZH2 [Exons 4,16,17,18]*** | *HNF1A* | *MEF2B* | *PARP2* | *REL* | *TNFAIP3* |
| *BARD1* | *CDKN2B* | *EZR* {Introns 9-11}* | ***HRAS [Exons 2,3]*** | *MEN1* | *PARP3* | ***RET [Exons 11,13-16, Introns 9,10,11]*** | *TNFRSF14* |
| *BCL2* | *CDKN2C* | *FAM46C* | *HSD3B1* | *MERTK* | *PAX5* | *RICTOR* | ***TP53*** |
| *BCL2L1* | *CEBPA* | *FANCA* | *ID3* | ***MET*** | *PBRM1* | *RNF43* | *TSC1* |
| *BCL2L2* | *CHEK1* | *FANCC* | ***IDH1 [Exon 4]*** | *MITF* | *PDCD1* | ***ROS1 [Exons 31,36-38,40, Introns 31-35]*** | *TSC2* |
| *BCL6* | ***CHEK2*** | *FANCG* | ***IDH2 [Exon 4]*** | *MKNK1* | ***PDCD1LG2*** | *RPTOR* | *TYRO3* |
| *BCOR* | *CIC* | *FANCL* | *IGF1R* | *MLH1* | ***PDGFRA [Exons 12,18]*** | *RSPO2* {Intron 1}* | *U2AF1* |
| *BCORL1* | *CREBBP* | *FAS* | *IKBKE* | ***MPL [Exon 10]*** | ***PDGFRB [Exons 12-21,23]*** | *SDC4* {Intron 2}* | ***VEGFA*** |
| *BCR* {Introns 8, 13, 14}* | ***CRKL*** | *FBXW7* | *IKZF1* | *MRE11A* | *PDK1* | *SDHA* | *VHL* |
| ***BRAF [Exons 11-18]*** | *CSF1R* | *FGF10* | *INPP4B* | *MSH2* | *PIK3C2B* | *SDHB* | *WHSC1* |
| ***BRCA1*** | *CSF3R* | *FGF12* | *IRF2* | *MSH3* | *PIK3C2G* | *SDHC* | *WHSC1L1* |
| ***BRCA2*** | *CTCF* | *FGF14* | *IRF4* | *MSH6* | ***PIK3CA [Exons 2,3,5-8,10,14,19,21 (Coding Exons 1, 2, 4-7, 9, 13,18,20)]*** | *SDHD* | *WT1* |
| *BRD4* | *CTNNA1* | *FGF19* | *IRS2* | *MST1R* | *PIK3CB* | *SETD2* | *XPO1* |
| *BRIP1* | ***CTNNB1 [Exon 3]*** | *FGF23* | *JAK1* | *MTAP* | *PIK3R1* | *SF3B1* | *XRCC2* |
| *BTG1* | *CUL3* | *FGF3* | ***JAK2 [Exons 14]*** | ***MTOR [Exons 19,30,39,40,43-45,47,48,53,56]*** | *PIM1* | *SGK1* | *ZNF217* |
| *BTG2* | *CUL4A* | *FGF4* | ***JAK3 [Exons 5,11,12,13,15,16]*** | *MUTYH* | *PMS2* | *SLC34A2* {Intron 4}* | *ZNF703* |
| ***BTK [Exons 2,15]*** | *CXCR4* | *FGF6* | *JUN* | *MYB* {Intron 14}* | *POLD1* | *SMAD2* |  |
| *C11orf30* | *CYP17A1* | ***FGFR1*** | *KDM5A* | ***MYC*** | *POLE* | *SMAD4* |  |
| *CALR* | *DAXX* | ***FGFR2 [Intron 17]*** | *KDM5C* | *MYCL* | *PPARG* | *SMARCA4* |  |
| *CARD11* | *DDR1* | ***FGFR3 [Exons 7, 9 (alternative designation exon 10), 14, 18, Intron 17]*** | *KDM6A* | ***MYCN*** | *PPP2R1A* | *SMARCB1* |  |
