# Supplementary material for: Clinical and analytical validation of FoundationOne Liquid CDx, a novel 324-Gene cfDNA-based comprehensive genomic profiling assay for cancers of solid tumor origin
Source: PLoS One. 2020 Sep 25;15(9):e0237802. doi: 10.1371/journal.pone.0237802 (PMC7518588; doi:10.1371/journal.pone.0237802)
Supplement: S2 Table — (DOCX) [file pone.0237802.s002.docx]

S2 Table. 37 cancer types represented in validation studies

| **Cancer Type** | **Unique Samples Included in Analytical Validation Studies** |
| --- | --- |
| Non-small cell lung carcinoma (NSCLC) | 85 |
| Breast | 70 |
| Prostate | 47 |
| Colorectal (CRC) | 43 |
| Unknown primary carcinoma (CUP) | 39 |
| Ovarian | 18 |
| Underspecified | 16 |
| Melanoma | 15 |
| Stomach | 12 |
| Cholangiocarcinoma | 10 |
| Pancreas | 8 |
| Esophagus | 5 |
| Biliary | 3 |
| Small cell lunch cancer | 3 |
| Thyroid | 3 |
| Gastrointestinal-neuro | 2 |
| Gastrointestinal stromal tumor (GIST) | 2 |
| Salivary gland | 2 |
| Anus | 1 |
| Bladder | 1 |
| Cervix | 1 |
| Diffuse large b-cell lymphoma (DLBCL) | 1 |
| Endocrine-neuro | 1 |
| Endometrial | 1 |
| Fallopian tube | 1 |
| Female-neuro | 1 |
| Head and neck | 1 |
| Liver | 1 |
| Myelodysplastic syndrome (MDS) | 1 |
| Soft tissue rhabdomyosarcoma | 1 |
| Potential myelodysplastic-myeloproliferative neoplasm (MDS-MPN) | 1 |
| Skin | 1 |
| T-prolymphocytic leukemia | 1 |
| Thymus | 1 |
| Unknown primary-neuro | 1 |
| Unknown primary gastrointestinal stromal tumor (GIST) | 1 |
| Uterus sarcoma | 1 |
