# Supplementary material for: Clinical and analytical validation of FoundationOne Liquid CDx, a novel 324-Gene cfDNA-based comprehensive genomic profiling assay for cancers of solid tumor origin
Source: PLoS One. 2020 Sep 25;15(9):e0237802. doi: 10.1371/journal.pone.0237802 (PMC7518588; doi:10.1371/journal.pone.0237802)
Supplement: S3 Table — (DOCX) [file pone.0237802.s003.docx]

S3 Table. Hit rate comparison between contrived and clinical specimens

| **n = 1,173 tests** | | | |
| --- | --- | --- | --- |
| **Alteration Type** | **Bait Set Region** | **Clinical Sample Targeted Level with ≥95% Hit Rate** | **Contrived Samples**  **Targeted Level with ≥95% Hit Rate** |
| Short Variants | Enhanced Sensitivity Region | 0.30% VAF^1^ | 0.35% VAF^1^ |
| Rearrangements | Enhanced Sensitivity Region | 0.20% VAF | 0.30% VAF |
| Copy Number Amplifications | NA | 5% TF | 5% TF |
| MSI | NA | 1% unstable loci | 0.8% unstable loci |
| bTMB (component indels) | NA | 1.5% VAF | 1.0% VAF |
| bTMB (component subs) | NA | 1.1% VAF | 1.0% VAF |

^1^ Mean LoD for substitutions and indels; VAF= variant allele fraction; TF = tumor fraction
