# Supplementary material for: Clinical and analytical validation of FoundationOne Liquid CDx, a novel 324-Gene cfDNA-based comprehensive genomic profiling assay for cancers of solid tumor origin
Source: PLoS One. 2020 Sep 25;15(9):e0237802. doi: 10.1371/journal.pone.0237802 (PMC7518588; doi:10.1371/journal.pone.0237802)
Supplement: S4 Table — (DOCX) [file pone.0237802.s004.docx]

S4 Table. Contrived samples platform LoD

| **Region** | **Alteration Sub-Type** | **N** | **Minimum VAF/TF^1^** | **1st Quantile** | **Mean VAF/TF^1^** | **Median VAF/TF^1^** | **3^rd^ Quantile** |
| --- | --- | --- | --- | --- | --- | --- | --- |
| Enhanced Sensitivity Region | Short Variants: Enhanced Sensitivity Region Total | 269 | 0.198% | 0.326% | 0.447% | 0.397% | 0.500% |
|  | Insertion/Deletion in non-repetitive region or a repetitive region of <=3 base pairs | 10 | 0.234% | 0.294% | 0.339% | 0.309% | 0.355% |
|  | Insertion/Deletion in a repetitive region of 4 to 6 base pairs | 23 | 0.282% | 0.365% | 0.546% | 0.482% | 0.560% |
|  | Insertion/Deletion in a repetitive region of >=7 base pairs | 6 | 0.326% | 0.481% | 0.663% | 0.578% | 0.820% |
|  | Substitution in a non-repetitive region or a repetitive region of <=7 base pairs | 229 | 0.198% | 0.329% | 0.437% | 0.393% | 0.489% |
|  | Substitution in a repetitive region of >7 base pairs | 1 | 0.317% | 0.317% | 0.317% | 0.317% | 0.317% |
| Standard Sensitivity Region | Short Variants: High Sensitivity Region Total | 595 | 0.401% | 0.702% | 0.879% | 0.822% | 0.976% |
|  | Insertion/Deletion in non-repetitive region or a repetitive region of <=3 base pairs | 18 | 0.455% | 0.682% | 0.832% | 0.871% | 1.00% |
|  | Insertion/Deletion in a repetitive region of 4 to 6 base pairs | 32 | 0.609% | 0.748% | 0.907% | 0.873% | 0.951% |
|  | Insertion/Deletion in a repetitive region of >=7 base pairs | 11 | 0.588% | 1.07% | 1.36% | 1.15% | 1.20% |
|  | Substitution in a non-repetitive region or a repetitive region of <=7 base pairs | 524 | 0.401% | 0.697% | 0.865% | 0.810% | 0.956% |
|  | Substitution in a repetitive region of >7 base pairs | 8 | 0.694% | 0.831% | 1.10% | 0.964% | 1.28% |
| Enhanced Sensitivity Region | Rearrangements | 7 | 0.199% | 0.257% | 0.508% | 0.366% | 0.474% |
| Enhanced/ Standard Sensitivity Region | Rearrangements | 1 | 0.284% | 0.284% | 0.284% | 0.284% | 0.284% |
| Standard Sensitivity Region | Rearrangements | 1 | 0.897% | 0.897% | 0.897% | 0.897% | 0.897% |
| NA | Copy Number Amplifications | 8 | 19.8% | 19.8% | 22.3% | 21.7% | 25.2% |
| NA | Copy Number Losses | 1 | 12.7% | 12.7% | 12.7% | 12.7% | 12.7% |

^1^VAF reported for short variant and rearrangement LoD, tumor fractoin reported for copy number amplification and copy number loss LoD
