# Supplementary material for: Clinical and analytical validation of FoundationOne Liquid CDx, a novel 324-Gene cfDNA-based comprehensive genomic profiling assay for cancers of solid tumor origin
Source: PLoS One. 2020 Sep 25;15(9):e0237802. doi: 10.1371/journal.pone.0237802 (PMC7518588; doi:10.1371/journal.pone.0237802)
Supplement: S5 Table — (DOCX) [file pone.0237802.s005.docx]

S5 Table: Reproducibility study results for a subset of clinically-actionable variants

| **Alteration** | **Variant Type** | **%VAF or %TF** | **Concordant Results** | **Reproducibility (%)** | **95% two-sided exact CIs (%)** |
| --- | --- | --- | --- | --- | --- |
| ALK-EML4 | Rearrangement | 0.64 | 24 of 24 | 100 | (85.75, 100) |
| ALK-EML4 | Rearrangement | 0.89 | 23 of 23 | 100 | (85.18, 100) |
| ALK-EML4 | Rearrangement | 1.39 | 24 of 24 | 100 | (85.75, 100) |
| ALK-NPM1 | Rearrangement | 0.64 | 24 of 24 | 100 | (85.75, 100) |
| ALK-NPM1 | Rearrangement | 0.40 | 18 of 23 | 78.26 | (56.3, 92.54) |
| ATM_5318delA | Short Variant | 0.77 | 24 of 24 | 100 | (85.75, 100) |
| ATM_5318delA | Short Variant | 1.04 | 23 of 23 | 100 | (85.18, 100) |
| ATM_6034_6035insCAGAAGTA | Short Variant | 0.86 | 23 of 23 | 100 | (85.18, 100) |
| ATM_8850+1G>A | Short Variant | 0.56 | 24 of 24 | 100 | (85.75, 100) |
| ATM-EXPH5 | Rearrangement | 1.13 | 24 of 24 | 100 | (85.75, 100) |
| BRAF_1790T>G | Short Variant | 0.42 | 22 of 23 | 95.65 | (78.05, 9.89) |
| BRAF_1790T>G | Short Variant | 0.85 | 24 of 24 | 100 | (85.75, 100) |
| BRAF_1798_1799GT>AA | Short Variant | 0.36 | 23 of 24 | 95.83 | (78.88, 9.89) |
| BRAF_1799T>A | Short Variant | 0.72 | 23 of 23 | 100 | (85.18, 100) |
| BRAF_1799T>A | Short Variant | 1.38 | 24 of 24 | 100 | (85.75, 100) |
| BRAF_1799T>A | Short Variant | 0.44 | 24 of 24 | 100 | (85.75, 100) |
| BRCA1_2338C>T | Short Variant | 1.11 | 24 of 24 | 100 | (85.75, 100) |
| BRCA1_2475delC | Short Variant | 0.61 | 24 of 24 | 100 | (85.75, 100) |
| BRCA1_2475delC | Short Variant | 0.93 | 24 of 24 | 100 | (85.75, 100) |
| BRCA1_2612C>TT | Short Variant | 0.51 | 23 of 23 | 100 | (85.18, 100) |
| BRCA1_68_69delAG | Short Variant | 0.66 | 24 of 24 | 100 | (85.75, 100) |
| BRCA1_P871fs*32 | Short Variant | 1.08 | 24 of 24 | 100 | (85.75, 100) |
| BRCA1-BRCA1 | Rearrangement | 0.87 | 24 of 24 | 100 | (85.75, 100) |
| BRCA2_3599_3600delGT | Short Variant | 0.58 | 24 of 24 | 100 | (85.75, 100) |
| BRCA2_3599_3600delGT | Short Variant | 0.92 | 24 of 24 | 100 | (85.75, 100) |
| BRCA2_4284_4285insT | Short Variant | 0.94 | 24 of 24 | 100 | (85.75, 100) |
| BRCA2_4284_4285insT | Short Variant | 1.26 | 23 of 23 | 100 | (85.18, 100) |
| BRCA2_5351delA | Short Variant | 1.22 | 24 of 24 | 100 | (85.75, 100) |
| BRCA2_5351delA | Short Variant | 1.85 | 24 of 24 | 100 | (85.75, 100) |
| BRCA2_5351delA | Short Variant | 1.07 | 23 of 23 | 100 | (85.18, 100) |
| BRCA2_5351delA | Short Variant | 2.24 | 24 of 24 | 100 | (85.75, 100) |
| BRCA2_5465_5466insA | Short Variant | 0.92 | 24 of 24 | 100 | (85.75, 100) |
| BRCA2_5465_5466insA | Short Variant | 1.19 | 23 of 23 | 100 | (85.18, 100) |
| BRCA2_799G>T | Short Variant | 0.5 | 22 of 24 | 91.67 | (73.0, 98.97) |
| BRCA2_8961_8964delGAGT | Short Variant | 1.07 | 24 of 24 | 100 | (85.75, 100) |
| BRCA2_9097_9098insA | Short Variant | 1.03 | 22 of 24 | 91.67 | (73.0, 98.97) |
| BRCA2_c.799G>T | Short Variant | 0.50 | 22 of 24 | 91.67 | (73.0, 98.97) |
| BRCA2_c.9097_9098insA | Short Variant | 0.71 | 5 of 23 | 21.74 | (7.46, 43.7) |
| BRCA2_c.9097_9098insA | Short Variant | 1.03 | 22 of 24 | 91.67 | (73.0, 98.97) |
| BRCA2_loss | Copy Number | 39.43 | 21 of 24 | 87.5 | (67.64, 97.34) |
| BRCA2-EDA | Rearrangement | 0.48 | 23 of 23 | 100 | (85.18, 100) |
| EGFR_2369C>T | Short Variant | 0.44 | 24 of 24 | 100 | (85.75, 100) |
| EGFR_2369C>T | Short Variant | 0.66 | 24 of 24 | 100 | (85.75, 100) |
| EGFR_2369C>T | Short Variant | 0.36 | 23 of 23 | 100 | (85.18, 100) |
| EGFR_2369C>T | Short Variant | 0.65 | 24 of 24 | 100 | (85.75, 100) |
| EGFR_2369C>T | Short Variant | 1.26 | 24 of 24 | 100 | (85.75, 100) |
| EGFR_2573T>G | Short Variant | 0.46 | 24 of 24 | 100 | (85.75, 100) |
| EGFR_2573T>G | Short Variant | 0.68 | 24 of 24 | 100 | (85.75, 100) |
| EGFR_2573T>G | Short Variant | 0.68 | 24 of 24 | 100 | (85.75, 100) |
| EGFR_2573T>G | Short Variant | 0.95 | 23 of 23 | 100 | (85.18, 100) |
| EGFR_2573T>G | Short Variant | 0.64 | 24 of 24 | 100 | (85.75, 100) |
| EGFR_2573T>G | Short Variant | 1.64 | 24 of 24 | 100 | (85.75, 100) |
| EGFR_E746_A750del | Short Variant | 0.51 | 24 of 24 | 100 | (85.75, 100) |
| EGFR_E746_A750del | Short Variant | 0.74 | 24 of 24 | 100 | (85.75, 100) |
| EGFR_E746_A750del | Short Variant | 0.93 | 24 of 24 | 100 | (85.75, 100) |
| EGFR_E746_A750del | Short Variant | 1.2 | 23 of 23 | 100 | (85.18, 100) |
| EGFR_E746_A750del | Short Variant | 0.51 | 23 of 23 | 100 | (85.18, 100) |
| EGFR_E746_A750del | Short Variant | 1.01 | 24 of 24 | 100 | (85.75, 100) |
| EGFR_E746_A750del | Short Variant | 0.34 | 22 of 22 | 100 | (84.56, 100) |
| ERBB2_amplification | Copy Number | 35.78 | 24 of 24 | 100 | (85.75, 100) |
| ERBB2_amplification | Copy Number | 39.79 | 24 of 24 | 100 | (85.75, 100) |
| ERBB2_amplification | Copy Number | 61.73 | 24 of 24 | 100 | (85.75, 100) |
| KRAS_182A>G | Short Variant | 0.53 | 24 of 24 | 100 | (85.75, 100) |
| KRAS_34_35GG>CT | Short Variant | 0.49 | 24 of 24 | 100 | (85.75, 100) |
| KRAS_35G>A | Short Variant | 0.89 | 24 of 24 | 100 | (85.75, 100) |
| KRAS_35G>A | Short Variant | 1.12 | 23 of 23 | 100 | (85.18, 100) |
| KRAS_38G>A | Short Variant | 0.55 | 24 of 24 | 100 | (85.75, 100) |
| KRAS_38G>A | Short Variant | 0.82 | 24 of 24 | 100 | (85.75, 100) |
| KRAS_38G>A | Short Variant | 0.57 | 23 of 23 | 100 | (85.18, 100) |
| KRAS_38G>A | Short Variant | 0.92 | 24 of 24 | 100 | (85.75, 100) |
| MET_2888-17_2888-3del15 | Short Variant | 1.17 | 24 of 24 | 100 | (85.75, 100) |
| MET_3005_3028+3>C | Short Variant | 1.67 | 24 of 24 | 100 | (85.75, 100) |
| MET_3029-1G>T | Short Variant | 0.3 | 21 of 23 | 91.3 | (71.96, 8.93) |
| MET_3933delC | Short Variant | 0.69 | 24 of 24 | 100 | (85.75, 100) |
| MET_3933delC | Short Variant | 0.96 | 24 of 24 | 100 | (85.75, 100) |
| MET_c.3029-1G>T | Short Variant | 0.21 | 15 of 24 | 62.5 | (40.59, 81.2) |
| MSI-H | BM | 0.91 | 24 of 24 | 100 | (85.75, 100) |
| MSI-H | BM | 1.33 | 24 of 24 | 100 | (85.75, 100) |
| MSI-H | BM | 3.07 | 24 of 24 | 100 | (85.75, 100) |
| MSI-H | BM | 6.45 | 24 of 24 | 100 | (85.75, 100) |
| NRAS_34G>T | Short Variant | 0.69 | 24 of 24 | 100 | (85.75, 100) |
| NRAS_34G>T | Short Variant | 0.96 | 24 of 24 | 100 | (85.75, 100) |
| NRAS_35G>A | Short Variant | 0.84 | 24 of 24 | 100 | (85.75, 100) |
| NRAS_c.35G>A | Short Variant | 0.48 | 19 of 23 | 82.61 | (61.22, 95.05) |
| NTRK1-MPRIP | Rearrangement | 0.49 | 16 of 23 | 69.57 | (47.08, 86.79) |
| NTRK1-MPRIP | Rearrangement | 0.69 | 21 of 24 | 87.5 | (67.64, 97.34) |
| NTRK1-TPM3 | Rearrangement | 0.3 | 24 of 24 | 100 | (85.75, 100) |
| NTRK1-TPM3 | Rearrangement | 0.4 | 24 of 24 | 100 | (85.75, 100) |
| NTRK1-TPM3 | Rearrangement | 8.48 | 22 of 24 | 91.67 | (73, 98.97) |
| NTRK2-N/A | Rearrangement | 1.85 | 23 of 24 | 95.83 | (78.88, 9.89) |
| NTRK2-N/A | Rearrangement | 2.03 | 23 of 24 | 95.83 | (78.88, 9.89) |
| NTRK3-ETV6 | Rearrangement | 0.32 | 23 of 24 | 95.83 | (78.88, 9.89) |
| NTRK3-ETV6 | Rearrangement | 0.59 | 23 of 24 | 95.83 | (78.88, 9.89) |
| NTRK3-ETV6 | Rearrangement | 26.33 | 24 of 24 | 100 | (85.75, 100) |
| PALB2_2422G>T | Short Variant | 0.47 | 23 of 23 | 100 | (85.18, 100) |
| PALB2_2422G>T | Short Variant | 0.92 | 24 of 24 | 100 | (85.75, 100) |
| PALB2_2724delA | Short Variant | 0.52 | 24 of 24 | 100 | (85.75, 100) |
| PALB2_2724delA | Short Variant | 0.74 | 24 of 24 | 100 | (85.75, 100) |
| PIK3CA_1624G>A | Short Variant | 0.89 | 24 of 24 | 100 | (85.75, 100) |
| PIK3CA_1633G>A | Short Variant | 0.45 | 24 of 24 | 100 | (85.75, 100) |
| PIK3CA_1633G>A | Short Variant | 0.66 | 24 of 24 | 100 | (85.75, 100) |
| PIK3CA_1633G>A | Short Variant | 0.5 | 24 of 24 | 100 | (85.75, 100) |
| PIK3CA_1634A>C | Short Variant | 0.52 | 24 of 24 | 100 | (85.75, 100) |
| PIK3CA_1634A>C | Short Variant | 0.7 | 23 of 23 | 100 | (85.18, 100) |
| PIK3CA_1637A>G | Short Variant | 0.49 | 22 of 23 | 95.65 | (78.05, 9.89) |
| PIK3CA_1637A>G | Short Variant | 0.92 | 24 of 24 | 100 | (85.75, 100) |
| PIK3CA_1645G>A | Short Variant | 0.48 | 24 of 24 | 100 | (85.75, 100) |
| PIK3CA_1645G>A | Short Variant | 0.73 | 24 of 24 | 100 | (85.75, 100) |
| PIK3CA_3140A>G | Short Variant | 0.41 | 23 of 23 | 100 | (85.18, 100) |
| PIK3CA_3140A>G | Short Variant | 0.76 | 24 of 24 | 100 | (85.75, 100) |
| PIK3CA_3140A>G | Short Variant | 1.04 | 24 of 24 | 100 | (85.75, 100) |
| PTEN_loss | Copy Number | 59.26 | 24 of 24 | 100 | (85.75, 100) |
| PTEN_loss | Copy Number | 46.89 | 24 of 24 | 100 | (85.75, 100) |
| PTEN_loss | Copy Number | 44.04 | 18 of 24 | 75 | (53.29, 90.23) |
| RET-CCDC6 | Rearrangement | 0.22 | 23 of 24 | 95.83 | (78.88, 9.89) |
| RET-CCDC6 | Rearrangement | 0.39 | 24 of 24 | 100 | (85.75, 100) |
| ROS1-CD74 | Rearrangement | 1.32 | 24 of 24 | 100 | (85.75, 100) |
| ROS1-EZR | Rearrangement | 1.3 | 24 of 24 | 100 | (85.75, 100) |
| ROS1-GOPC | Rearrangement | 0.91 | 22 of 24 | 91.67 | (73.0, 98.97) |
| ROS1-GOPC | Rearrangement | 0.35 | 20 of 23 | 86.96 | (66.41, 97.22) |
| ROS1-SLC34A2 | Rearrangement | 1.03 | 24 of 24 | 100 | (85.75, 100) |
| ROS1-SLC34A2 | Rearrangement | 1.36 | 23 of 23 | 100 | (85.18, 100) |

Note: VAF (variant allele frequency) used for substitutions, indels, and rearrangements; TF (tumor fraction) used for copy number alterations
