# Supplementary material for: Clinical and analytical validation of FoundationOne Liquid CDx, a novel 324-Gene cfDNA-based comprehensive genomic profiling assay for cancers of solid tumor origin
Source: PLoS One. 2020 Sep 25;15(9):e0237802. doi: 10.1371/journal.pone.0237802 (PMC7518588; doi:10.1371/journal.pone.0237802)
Supplement: S6 Table — (DOCX) [file pone.0237802.s006.docx]

S6 Table: Precision of bTMB

| **Sample** | **Mean bTMB score (mut/Mb)** | **Reproducibility** | |
| --- | --- | --- | --- |
|  |  | **SD** | **%CV** |
| Sample 1 | 6.955 | 0.6487 | 9.3% |
| Sample 2 | 12.1175 | 1.9831 | 16.4% |
| Sample 3 | 13.1167 | 1.1071 | 8.4% |
| Sample 4 | 13.2754 | 2.0682 | 15.6% |
| Sample 5 | 26.3421 | 1.942 | 7.4% |
| Sample 6 | 203.0379 | 12.1974 | 6.0% |

bTMB = blood tumor mutational burden; mut/Mb = mutations/megabase; SD = standard deviation; CV = coefficient of variation
