# Supplementary material for: Clinical and analytical validation of FoundationOne Liquid CDx, a novel 324-Gene cfDNA-based comprehensive genomic profiling assay for cancers of solid tumor origin
Source: PLoS One. 2020 Sep 25;15(9):e0237802. doi: 10.1371/journal.pone.0237802 (PMC7518588; doi:10.1371/journal.pone.0237802)
Supplement: S7 Table — (DOCX) [file pone.0237802.s007.docx]

S7 Table. Confirmation of LoD and precision in cfDNA specimens

| **Target Alteration** | **Mean VAF/TF/unstable loci Tested** | **Reproducibility (95% CI)** |
| --- | --- | --- |
| *BRAF* V600K | 0.36% VAF | 95.8 (78.88, 99.89) |
| *MET* exon 14splice site 3005_3028+3>C | 1.67% VAF | 100 (85.75, 100) |
| *ATM I2012fs*4* | 0.86% VAF | 100 (85.18, 100) |
| *ATM splice site 8850+1G>A* | 0.56% VAF | 100 (85.75, 100) |
| *ERBB2* CNA | 61.73% TF | 100 (85.75, 100) |
| *KRAS* G12L | 0.49% VAF | 100 (85.75, 100) |
| *KRAS* Q61R | 0.53% VAF | 100 (85.75, 100) |
| *MET* exon 14 splice site 2888-17_2888-3del15 | 1.17% | 100 (85.75, 100) |
| MSI-High | 6.45% unstable loci | 100 (85.75, 100) |
| *PIK3CA* E542K | 0.89% VAF | 100 (85.75, 100) |
| *PIK3CA* H1047R | 1.04% VAF | 100 (85.75, 100) |
| *PTEN* Loss | 46.89 TF | 100 (85.75, 100) |
| *BRCA2-* *EDA* Truncation | 0.48% VAF | 100 (85.18, 100) |
| *BRCA1* Rearrangement | 0.87% VAF | 100 (85.75, 100) |
| *ROS1-EZR* Rearrangement | 1.3% VAF | 100 (85.75, 100) |
|  |  |  |
| *MSI-High* | 3.07% unstable loci | 100 (85.75, 100) |
| *ALK-EML4* Rearrangement | 1.39 %VAF | 100 (85.75, 100) |
| *ATM-EXPH5* Truncation | 1.13% VAF | 100 (85.75, 100) |
| *BRCA1* Q780* | 1.11%VAF | 100 (85.75, 100) |
| *BRCA1 E23fs*17* | 0.66% VAF | 100 (85.75, 100) |
|  |  |  |
| *BRAF* V600E | 0.44% VAF | 100 (85.75, 100) |
| *EGFR* T790M | 1.26% VAF | 100 (85.75, 100) |
| *EGFR* L858R | 1.64% VAF | 100 (85.75, 100) |
| *ROS1-CD74* Fusion | 1.32% VAF | 100 (85.75, 100) |
|  |  |  |
| *EGFR* L858R | 0.64% VAF | 100 (85.75, 100) |
| *BRCA2* S2988fs*12 | 1.07% VAF | 100 (85.75, 100) |
| *PIK3CA* E545K | 0.5% VAF | 100 (85.75, 100) |
| *EGFR E746_A750del* | 0.34% VAF | 100 (84.56, 100) |

VAF = variant allele frequency; TF = tumor fraction
