# Supplementary material for: Clinical and analytical validation of FoundationOne Liquid CDx, a novel 324-Gene cfDNA-based comprehensive genomic profiling assay for cancers of solid tumor origin
Source: PLoS One. 2020 Sep 25;15(9):e0237802. doi: 10.1371/journal.pone.0237802 (PMC7518588; doi:10.1371/journal.pone.0237802)
Supplement: S8 Table — (DOCX) [file pone.0237802.s008.docx]

S8 Table. Comparison of FoundationOne Liquid CDx with the reference assay for the detection of *PIK3CA* alterations

|  | | **Reference Assay** | | | | |  |
| --- | --- | --- | --- | --- | --- | --- | --- |
|  |  | **Positive** | **Negative** | **Not Evaluable** | **Missing** | **Total** |  |
| **FoundationOne Liquid CDx** | **Positive** | 165 | 20 | 2 | 1 | 188 | PPA_:_ 89.19% [83.80%, 93.27%] |
|  | **Negative** | 5 | 222 | 1 | 2 | 230 | NPA: 97.80% [66.40%, 78.37%] |
|  | **Not Tested** | 29 | 65 | 2 | 0 | 96 |  |
|  | **Not Evaluable** | 6 | 35 | 0 | 0 | 41 |  |
|  | **Total** | 205 | 342 | 5 | 3 | 555 |  |
|  | | PPA: 97.06% [93.27%, 99.04%] | NPA: 91.74% [87.52%, 94.88%] |  |  |  | OPA: 93.93% [91.17%, 96.04%] |

PPA = positive percent agreement; NPA = negative percent agreement; OPA = overall percent agreement
